# The potential for selective pharmacological therapies through biased receptor signaling

**DOI:** 10.1186/2050-6511-13-3

**Published:** 2012-08-13

**Authors:** Terry Kenakin

**Affiliations:** 1 Department of Pharmacology, University of North Carolina School of Medicine, 120 Mason Farm Road, Room 4042 Genetic Medicine Building, CB# 7365, Chapel Hill, NC, 27599-7365, USA

## Abstract

The discovery that not all agonists uniformly activate cellular signaling pathways (biased signaling) has greatly changed the drug discovery process for agonists and the strategy for treatment of disease with agonists. Technological advances have enabled complex receptor behaviors to be viewed independently and through these assays, the bias for an agonist can be quantified. It is predicted that therapeutic phenotypes will be linked, through translational studies, to quantified scales of bias to guide medicinal chemists in the drug discovery process.

## Introduction

Agonists constitute a major class of therapeutic drug and pose special problems with respect to the drug discovery process. Specifically, agonist activity is intimately tied to the sensitivity of the tissue in which the activity is measured therefore the obvious measures of agonist activity (i.e. potency and maximal response) can be complex and certainly are system dependent. As in most cases, new therapeutic entities are discovered, optimized and characterized in test systems, rarely the therapeutic one. Therefore, system-independent reliable scales to describe agonist activity are critical in the process of identifying agonist drug candidates. A pharmacologic workhorse for the quantification of agonist activity has been the agonist potency ratio. This tool compares equal responses via null methods to cancel tissue effects to yield ratios that depend only on agonist affinity and efficacy. Since these are unique properties of the molecules, these ratios can be system independent and invaluable as predictors of activity in therapeutic systems. Potency ratios are applicable only when comparing full agonists and cannot be applied to comparisons of full to partial agonist activity. However, other techniques that identify system independent measures of agonist activity can be estimated through tools such as the Black/Leff operational model; this characterizes the affinity (as K_A_^-1^ where K_A_ is the equilibrium dissociation constant of the agonist-receptor complex) and τ (where τ is the agonist intrinsic efficacy and sensitivity to agonist of the tissue- *vide infra*) [1]. Thus estimates of τ and K_A_ become the system independent measures of agonism.

The use of these techniques to compare whole cell activity for drug discovery depends on an important assumption, namely that the function linking receptor occupancy and tissue response be ***monotonic*** (i.e. that there be only one value of y (response) for every x (concentration of drug)). Under these circumstances, ratios of activity seen for whole cell response should accurately mirror ratios of the initial stimulus that the agonist gives to the system (the molecular properties of affinity and efficacy). A further basic assumption in the use of whole cell relative activities of agonism is that all agonists stabilize a common active state of the receptor to generate response. In the absence of evidence to the contrary, this is the most parsimonious model of agonism and it has been operative for 50 years in pharmacology. However, over the past 20 years there have been a number of papers published that suggest that stimulus–response coupling is more complex [2-9] and that whole cell potency ratios for agonists can be variable. The basis for these ideas stem from examples where multiple responses are measured from a single receptor type showing that some agonists produce pleiotropic responses and others do not. As a preface to the discussion of the mechanism by which this can occur at the receptor level, it is useful to consider a subset of these examples where agonists demonstrate apparently selective pathway agonism because of the efficiency of stimulus-coupling in the cell.

### Cell system bias

**Figure 1:**
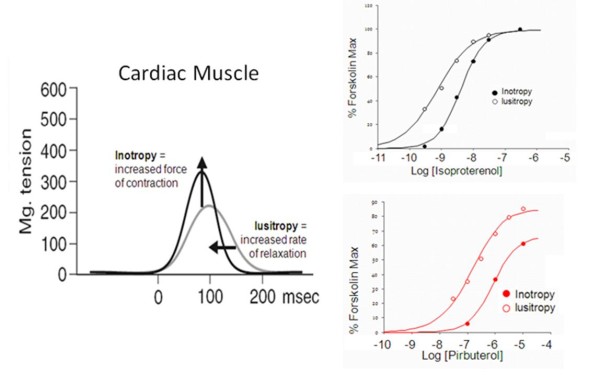
**Effects of isoproterenol and pirbuterol on rat atrial isometric contraction peak height (inotropy; filled circles) and rate of relaxation (lusitropy; open circles).** The concentration-response curve for lusitropy is shifted 3-fold to the left of the curve for inotropy indicating a difference in the intrinsic coupling efficiencies of the β-adrenoceptor to these two physiological processes. Data redrawn from [10].

The efficiency of coupling of various cellular pathways is probably tailored to the needs of the cell. For example, β-adrenoceptor activation in the rat atrium produces myocardial inotropy (increase force of isometric contraction) and lusitropy (increased rate of relaxation) and it can be seen that dose–response curves for lusitropic effects are shifted by a factor of three to the left of those for inotropic effects [10]-see Figure 1. This would be consistent with a condition whereby lusitropy requires a lower level of cyclic AMP elevation than does inotropy. The relevant point to this discussion is that this cellular bias with respect to signaling is a property of the tissue (not any specific difference in the efficacy of the agonists) and thus it is a condition that will be true for all agonists in the tissue; these effects will be referred to as ‘*system bias’*. Differences in the sensitivity of pathways becomes even more pronounced when different assays are compared. For example, it is generally true that enzyme complementation assays for β-arrestin effects are considerably less sensitive than second messenger assays such as cyclic AMP elevation. This can lead to a number of apparently ‘selective’ agonist effects whereby weak agonists can produce activation of only the most efficiently coupled pathway; this effect will be referred to as ‘*observation bias’*. These ‘strength of signal’ profiles are not necessarily examples of ligand bias and should not be considered as such.

Every pharmacologic experiment concerning agonism will be subject to system and observation bias but these effects can be cancelled through null procedures whereby agonists are compared to each other within a given signaling pathway. In addition, these types of bias generally are not useful therapeutically since all agonists are subject to their effects and the extent of the effect is linked to the nature of the system (i.e. species, organ, experimental conditions etc.). True ligand bias is superimposed upon these effects to produce a ligand-specific agonism that will translate to *in vivo* systems and give phenotypically unique profiles of agonism.

### Ligand directed signaling

Within the time period where variance of experimental data from theoretical prediction were reported there began to appear cases where a simple strength of signal mechanism could not account for differences in agonist response. It should be noted that the important advancement in pharmacology that allowed these effects to be studied was (and is) the availability of multiple assays to view receptor behavior. Thus, for example, instead of inferring effects on receptor desensitization and internalization from the disappearance of agonist response, these effects could be viewed independently with imaging assays. When this was done it was seen that internalization did not always parallel agonism and in fact, could be shown to be an independent phenomenon [11,12].

The major type of observation to suggest that agonists possessed the innate property of controlling cellular pathway stimulation is the demonstration that the relative potencies of agonists actually ***reverse order*** when two pathways controlled by the same receptor are observed. For example, in LLC-PK1 cells transfected with PACAP receptors the relative potencies of the PACAP analogues PACAP_1-27_ and PACAP_1-38_ for increasing cellular cyclic AMP and inositol phosphate were determined. It was observed that the two agonists reverse their relative order of potency for the two signaling pathways mediated by the same receptor (i.e. the relative potency for cyclic AMP is PACAP_1-27_ > PACAP_1-38_ and PACAP_1-27_ < PACAP_1-38_ for inositol phosphate production) [13]. Similarly, the relative potency of Eel and Porcine calcitonin reversed order when HEK cells were enriched with G_αs_ through transduction [14]. In these cases, a strength of signal mechanism cannot account for the difference in potency ratio and another hypothesis is needed.

**Figure 2:**
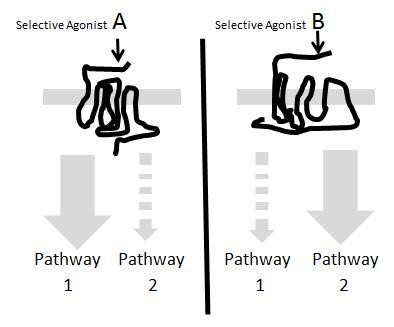
**Molecular mechanism for agonist-directed biased signaling.** Agonist A stabilizes a conformation of the receptor that preferably activates pathway 1 while agonist B stabilizes a receptor conformation that preferably activates pathway 2.

This type of divergent data for relative agonist potency clearly suggested that the binding of different agonists leads to the production of different receptor species with varying preference for signaling proteins, i.e. different agonists produce different receptor active states; the first proposal of this mechanism labeled the effect ‘stimulus-trafficking’ [15]-see Figure 2. Subsequently, a number of pharmacologic approaches utilizing cellular agonist response were used and data consistent with ligand-specific receptor active state formation were reported (for reviews see [16-20]). Notably, studies with dopamine agonists [3,21] and serotonin [22] clearly showed the effect; the most common terms utilized to describe these effects over this period were agonist ‘bias’ or ‘functional selectivity’. In addition to cellular pharmacologic studies, agonist formation of different receptor states has been shown through chemical and structural studies with a number of techniques (see [23-38]).For example, ^19^ F-NMR studies show the adoption of different β_2_-adrenoceptor conformations with binding of different ligands [39]. Therefore, in addition to system bias for any two pathways in the cell (or any two functional response readouts) a ligand bias can be imposed.

### How signaling bias alters ligand pharmacology

True ligand bias denotes that the chemical structure of the molecule has coded within it the information needed to route stimulus to selected signaling proteins in the cell; this is done through the stabilization of a unique receptor conformations. When controlled through medicinal chemistry, this phenomenon has the potential to improve the signaling profiles of agonists and antagonists for therapeutic benefit. The key to achieving this type of control is to have assays that can detect and quantify selective pathway signaling. For example, *in vitro* assays that measure second messenger production (eg. cyclic AMP from G_αs_ activation of adenylate cyclase) and the association of receptor with β-arrestin (Bioluminescent Resonance Energy Transfer, BRET) can be used to separately assess the ability of a ligand to activate the pathways. The other requirement is a quantitative scale by which pharmacologists and medicinal chemists can gauge the effect of changing molecular structure on the bias of a given chemical scaffold. The key to such a scale is that it be intimately associated with the specific pathway being assessed, i.e. as discussed previously, parameters that characterize the receptor species interacting with the signaling molecules must be determined. As a preface to the discussion of system independent parameters of agonism, it is useful to describe the theoretical context of the model for agonism, namely the Black/Leff operational model [1]. In this model, response is controlled by the affinity of the agonist (denoted as K_A_^-1^ where K_A_ is the equilibrium dissociation constant of the agonist-receptor complex) and efficacy denoted by τ where τ = [R_t_ (membrane receptor density) divided by K_E_, the virtual Michaelis-Menten constant for the interaction of the agonist-bound receptor as it interacts with the cell where the cell is defined as a virtual enzyme. The efficacy term τ thus describes both the intrinsic efficacy of the agonist (i.e. the power of the molecule to induce response) and the sensitivity of the system to return response (number of responding units [receptors] and the efficiency of receptor coupling to stimulus–response mechanisms in the cell). Response is thus given by the equation:

(1) $$\text{Response=}\frac{{\left[\text{A}\right]}^{\text{n}}\tau^{\text{n}}{\text{E}}_{\text{m}}}{{\left[\text{A}\right]}^{\text{n}}\tau^{\text{n}}+{\left(\left[\text{A}\right]+{\text{K}}_{\text{A}}\right)}^{\text{n}}}$$

where n is the Hill coefficient for the agonist concentration-response curve. Since receptors are allosteric proteins, these parameters must involve the constants that define the interactions of the agonist (termed the allosteric modulator of the effect) and the guest (in this case the signaling molecule) with the receptor. The former parameter is the affinity of the agonist for the receptor (K_A_) which, for allosteric proteins such as receptors, is a conditional parameter. Affinity must be included in the parameter estimate since allosteric mechanisms dictate that K_A_ will be different for the agonist-receptor pair for different signaling molecules. This has been shown in structural and binding studies for β_2_-adrenoceptors [40], κ-opioid receptors [41], and ghrelin receptors [42]. The parameter τ is a characteristic ‘efficacy’ of the resulting agonist-receptor complex. Therefore the minimal theoretically sound unit to denote agonist power to produce activation of any given cellular pathway is τ/K_A_ (generally utilized as the log normal parameter Log (τ/K_A_)). While this parameter can be estimated by fitting data to equation 1, it also can be shown that for systems where n = 1, Log (τ/K_A_) values reduce to Log (RA) values where RA refers to ‘Receptor Activity’ indices defined by Ehlert and colleagues [43] as (maximal response)/EC_50_ for agonism (where EC_50_ is the molar concentration producing 50 % maximal response to the agonist). Log (RA) values are the most simple index of agonism and take into account both the sensitivity of the system and the intrinsic efficacy of the agonist. Therefore, the relative Log (τ/K_A_) (or for n = 1 cases Log (RA)) values can be evaluated for a series of agonists in a given pathway to rank the relative intrinsic efficacy of the agonists for that pathway. Comparison to the same reference agonist (usually the natural endogenous agonist) for different pathways can then be used to compare the relative power of the agonists to activate different pathways [44]. Theoretically, providing the log (τ/K_A_) values represent direct measurement of modulator (agonist)-conduit (receptor)-guest (signaling pathway complexes, they will be system independent measures of agonism [16,44]. Of course where response is measured from points beyond this complex (i.e. where the cell mixes and matches components of response), even Log (τ/K_A_) values become system dependent and less useful as measures of agonist bias.

### Therapeutic implications of biased signaling

The realization that the signaling profiles of agonists may be subject to modification has led to an explosion of proposals in the literature for improved agonists (and antagonists). These are based on data to show that some but not all signaling produced by agonists is beneficial to the host organ. For instance, opioids are valuable analgesics but also can produce respiratory depression. Insofar as respiratory depression can be linked to activation of β-arrestin [45]), an opioid agonist that stimulates opioid pathways without promoting receptor/β-arrestin interaction would be predicted to be a superior therapy [46-48]. Treatment with synthetic orthosteric agonists also precludes receptor occupancy by the endogenous agonist and this becomes an important aspect of the *in vivo* profile of biased ligands. For instance, in heart failure, blockade of angiotensin receptors precludes damaging angiotensin-mediated vasoconstriction. Treatment with a biased angiotensin antagonist such as TRV120027; Sar-Arg-Val-Tyr-Ile-His-Pro-D-Ala-OH blocks angiotensin but also promotes beneficial effects of β-arrestin activation (stimulation of p42/44 mitogen-activated protein kinase, Src, and endothelial nitric-oxide synthase phosphorylation ) and it is predicted that this will lead to a therapeutic advantage [49-53]. Initial data support this conclusion. In rats, blockade of endogenous angiotensin with conventional angiotensin receptor antagonists losartan and telmisartan, leads to reduced mean arterial pressure but also a decrease in cardiac performance. In contrast, treatment with TRV120027 does not decrease but rather increases cardiac performance and preserves cardiac stroke volume [54]. Similarly, in canine heart failure models TRV120027 produces cardiac unloading actions but preserves renal function resulting in a predicted novel strategy for the treatment of heart failure [55]. It should be noted that while stabilization of receptor conformation is the most commonly proposed mechanism for biased ligand effects, there are potentially other mechanisms that may be operative, especially with respect to peptide ligands such as TRV120027. For example, it has been shown that differential dissociation of the peptides RANTES and AOP-RANTES from internalized CCR5 receptors causes differences in the recycling of receptors back to the cell surface upon internalization. Specifically, the lack of dissociation of AOP-RANTES from the receptor in the acidic cytoplasmic environment leads to selective CCR5 endosomal destruction as compared to rapid recycling with RANTES [56]. In general, biased signaling has been proposed to be potentially useful in a host of diseases including hyperlipidemia (GPR109 receptors [57,58]), heart failure (β-adrenoceptors [59-61]), some neuropsychiatric/ neurodegenerative disorders (histamine H_1_ receptors [62]), hypertension (α_2_-adrenoceptors [63]), hypothyroidism (TSR [64]),schizophrenia (dopamine D_2_ receptors [65-67]), small-cell lung cancer (GRPR/vasopressin [68]), osteoporosis (PTH receptors [69-71]), parkinsonism (dopamine D1 receptors [72]), diabetes (GLP-1 receptors [73]), addiction, psychosis and depression (5-HT receptors [74,75]).

The consideration of biased signaling in various therapeutic areas has been introduced into pharmacology through various means including theoretical predictions based on known signaling components of cells and from studies in gene knockout animals (eg. knockout animals for β-arrestin-1 [73], β-arrestin-2 [45], p90 ribosomal S6 kinase [76]). However, there are numerous instances where it still is not yet possible to predict which type of signaling bias may represent a superior therapy. In these cases, empirical testing of exemplar molecules in animal models is a way forward; these approaches have generally led to a revolution in the strategy for new drug discovery.

### The impact of signaling bias on drug discovery

**Figure 3:**
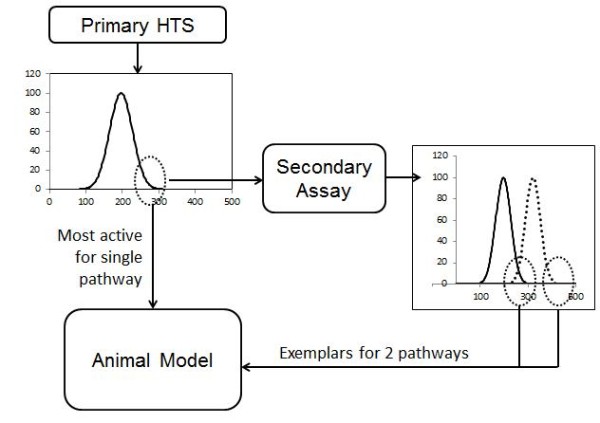
**Impact of agonist-directed stimulus on drug screening.** Canonical strategies for high throughput (HTS) screening pass the most active molecules from the HTS (represented by the dextral tail of the Boltzman distribution representing the best responses in the HTS) into secondary assays and animal models for further testing. To detect bias in signaling, the active molecules from the HTS are all tested in another functional assay for another signaling pathway and the most active molecules from that assay pooled with the actives from the HTS for further testing. The most active molecules in the secondary biased assay often are not the most active in the HTS thus a spectrum of agonists of differing stimulus bias is tested in animal models.

When it was assumed that new synthetic ligands mimic natural endogenous agonists in their quality of efficacy (the pathways they activated in the cell) and only differed in the quantity of efficacy they possessed, a single robust high throughput screen (HTS) with a suitably sensitive readout of cellular response was theoretically adequate to find agonists. The discovery that this is not the case and that some agonists activate selected pathways more than others destroys this assumption. Specifically, a biased agonist may have weak activity in one assay tailored to measure a given pathway but a much stronger activity for another pathway not measured by the screening assay. This compels the testing of agonists in multiple pathways, an idea at odds with the obvious resource constraints of discovery efforts. A fruitful compromise may be to test the discovered active molecules from an HTS in other assays designed to measure another pathway. The testing of screening actives in another pathway assay would allow the detection of texture in agonism, i.e. extremes in stimulation profiles could then be taken into animal models to possibly detect unique phenotypes in these models-see Figure 3.

The idea that agonists may bias the stimulus they give to cells is relatively new and as such, may be thought to be somewhat rare. However, an alternative view which considers that receptors can form numerous states (some active with respect to signaling) and that ligands interact with an ‘ensemble’ of different conformations, predicts a vast array of bias for different agonists [77-82]. Therefore the agonist-stabilized ensemble is the result of an array of conformations stabilized through binding governed by the affinities of the agonist for particular conformations. This idea suggests that it would be unlikely that any two agonists would have identical bias with respect to an array of signaling molecules. Under these circumstances, the high probability that ligands will not have identical affinities for a large number of conformations predicts that ligands will in essence stabilize a nearly unique conformational ensemble and this, in turn, would go on to activate a nearly unique cadre of stimulus cascades in the cell.

## Conclusions

The idea that new agonists may well produce receptor conformations that activate signaling proteins in a biased manner forces pharmacologists to rethink their concepts of agonism, i.e. the most potent and efficacious agonist may not be the best option for therapy. These ideas have extended the field of target validation beyond target type to which signaling pathway mediated by that target type is the relevant ‘target’ for therapy. It will be most interesting to see if phenotypic signaling determined from in vitro assays translates to unique therapeutic phenotypes in vivo. The various discovery efforts with biased ligands presently in progress should provide answers to this question within the next few years.

## Competing interests

The authors declare that they have no competing interests.

## Author contribution

TPK wrote the article.

## Pre-publication history

The pre-publication history for this paper can be accessed here:

http://www.biomedcentral.com/2050-6511/13/3/prepub
